# HIV self-testing: what GetaKit can tell us about Canada’s $8 million one-time investment

**DOI:** 10.17269/s41997-023-00768-3

**Published:** 2023-04-13

**Authors:** Patrick O’Byrne, Alexandra Musten

**Affiliations:** School of Nursing, Faculty of Health Sciences, University of Ottawa, 451 Smyth Road, Ottawa, ON K1H 8M5 Canada

**Keywords:** HIV, Self-testing, Canada, Impact fraction model, GetaKit, VIH, autodépistage, Canada, modèle impact fraction, GetaKit

## Abstract

At the 16^th^ International AIDS Conference in Montreal, Canada’s Federal Health Minister announced that the Government of Canada will invest $17 million to increase access to HIV testing, $8 million of which would be used to purchase and distribute HIV self-tests. While HIV testing, and subsequent diagnoses, is a critical first step to achieving the updated UNAIDS goals of 95-95-95, testing on its own does not guarantee linkage to treatment or prevention services. In other words, it does not alone guarantee progress toward the 95-95-95 goals. GetaKit, Canada’s first HIV self-test mail-out project, has demonstrated that a preliminary risk-assessment consistent with US CDC and PHAC screening guidelines ensures targeted uptake among communities most affected by HIV, thus minimizing the risk of false positive results and poor positive predictive values. Furthermore, HIV self-testing must link not only individuals with positive results to treatment, but also persons with negative results to pre-exposure prophylaxis (PrEP) along with re-testing as required. However, both access to treatment and PrEP remain inconsistently available across Canada. Therefore, while this one-time investment of funding to increase HIV testing is encouraging, without clear instructions as to who should be prioritized for testing and definitive next steps to ensure that individuals are successfully linked to care, Canada risks wasting resources, further exacerbating pre-existing inequities.

## Résumé

Lors de la 16e Conférence internationale sur le sida qui s’est tenue à Montréal, le ministre fédéral de la Santé a annoncé que le gouvernement du Canada investirait 17 millions de dollars pour améliorer l’accès au dépistage du VIH, dont 8 millions pour l’achat et la distribution d’autotests de dépistage. Bien que le dépistage du VIH et les diagnostics qui en découlent constituent une première étape essentielle pour atteindre les objectifs actualisés de l’ONUSIDA (95-95-95), le dépistage en lui-même ne garantit pas l’accès à des services de traitement ou de prévention. En autres mots, il ne garantit en lui-même le progrès vers l’objectif 95-95-95. GetaKit, le premier projet canadien d’envoi postal d’autotests de dépistage du VIH, a démontré qu’une évaluation préliminaire des risques conforme aux directives de dépistage des CDC américains et de l’ASPC garantit une participation ciblée au sein des communautés les plus touchées par le VIH, minimisant ainsi le risque de résultats faussement positifs et de valeurs prédictives positives médiocres. En outre, l’autodépistage du VIH doit permettre non seulement aux personnes dont les résultats sont positifs de suivre un traitement, mais aussi aux personnes dont les résultats sont négatifs de bénéficier d’une prophylaxie pré-exposition (PrEP) et d’un nouveau test, le cas échéant. Cependant, l’accès au traitement et à la PrEP n’est pas uniformément disponible au Canada. Par conséquent, bien que cet investissement ponctuel de fonds pour augmenter le dépistage du VIH soit encourageant, en l’absence d’instructions claires sur les personnes à tester en priorité et sur les prochaines étapes à suivre pour s’assurer que les personnes sont bien dirigées vers les soins nécessaires, le Canada risque de gaspiller des ressources, ce qui ne fera qu’exacerber les inégalités déjà existantes.

On December 1, 2020, the UNAIDS (2020) changed its 90-90-90 HIV targets to 95-95-95, updating its aims to have 95% of persons living with HIV diagnosed, 95% of those who are diagnosed linked to care, and 95% of those in care achieving undetectable viral loads. In Canada, in 2020, an estimated 10% of HIV-positive persons remained undiagnosed, signalling success with the previous aims and work to do for the new targets (Public Health Agency of Canada (PHAC), 2022a). Also highlighting the need for ongoing work is that new HIV infections continue to disproportionately affect members of the same populations (henceforth referred to as *priority populations*), including gay, bisexual, trans, and other men who have sex with men; members of Indigenous communities; persons of African, Caribbean, and Black ethnicities; and people who use injection drugs (PHAC, 2022a).

The latest addition to our armamentarium to achieve the 95-95-95 goals in Canada is self-testing, which Health Canada approved in November 2020 (CATIE, 2020). Preceding that, with special access program approval, GetaKit launched Canada’s first at-home HIV self-testing project in Ontario in July 2020 (O’Byrne et al., 2021a). As an automated version of the United States Centers of Disease Control (CDC, 2021) STI risk assessment, the GetaKit system imputes participants’ need for HIV testing to ensure self-tests are distributed to persons with risk factors for HIV acquisition (O’Byrne et al., 2021b). Since its inception, over 6000 persons have obtained HIV self-tests from GetaKit, of whom 27% denied any form of prior HIV testing and 81% were members of the HIV priority populations. GetaKit has a positivity rate of 0.4%, compared to an overall HIV positivity rate in Ontario of 0.1% (OHESI, 2022). Some international data support that HIV self-testing yields positivity rates that exceed other testing modalities (Johnson et al., 2021).

It is thus unsurprising that the Public Health Agency of Canada (2022b) released millions of dollars worth of HIV self-tests in Canada, with these being distributed by AIDS Service Organizations (ASOs). Through an online ordering system, agencies across Canada can order and distribute free HIV self-tests to their clients and within their communities. Anyone who accesses these ASOs can then obtain up to five free HIV self-tests, and are encouraged to distribute these additional kits to others within their social and sexual networks. While the hope is that these HIV self-tests will be used and distributed onward by members of the HIV priority populations, there are no restrictions regarding who can order these tests or how many times they can do so.

While self-testing and Canada’s emphasis of HIV self-testing fulfills Wilson and Junger’s (1968) principles of screening (in that, the health condition is important, the test is accurate, there is treatment), the implementation of self-testing requires reflection, as a recent randomized controlled trial did not identify differences in positivity rates for self-testing versus serology, but did find that self-testing corresponded with more testing (Rodger et al., 2022). That is, self-testing used more resources without increasing diagnoses. This suggests that, while many studies often reveal that HIV self-testing corresponds with positivity rates that exceed traditional testing options (see Johnson et al., 2021), this outcome may not exist when both self-testing and traditional forms of testing are made readily available. Promoting equitable and safe access to HIV serology may therefore yield the same outcomes, at a lower cost, with established linkage to care options.

**Fig. 1:**
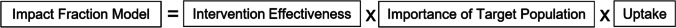
The impact fraction model

Considering Canada’s effort to distribute HIV self-tests in light of uncertainties within the extant knowledge about the real-world effectiveness of these tests, we employed the *impact fraction model* to guide our analysis. This model is useful because it identifies three questions to consider when designing, implementing, or evaluating interventions: (1) Is the intervention effective?; (2) Does the target population account for a large burden of the health condition?; and (3) Does the target population use the intervention? (Aral et al., 2007) (Fig. 1).

In using the impact fraction model (Aral et al., 2007) to guide our analysis, the first question is about effectiveness, for which HIV self-testing must be evaluated on two fronts. First, it is important to consider the performance metrics of the test. In Canada, the only approved HIV self-test is the bioLytical INSTI®, which has a published sensitivity of 99.8% and specificity of 99.5% (bioLytical, 2023). A controlled validation study that occurred in clinical settings in Canada also identified an invalid rate of 5.6% and that another 2.7% of participants were unable to interpret their results, meaning that 8.3% of the participants in that study did not obtain usable results (Galli et al., 2021). Real-world data from GetaKit, moreover, showed an invalid rate that started at 25% and decreased to 8% once we began giving participants additional supports and information about how to do the test (O’Byrne et al., 2022). Our interpretation is that, while the INSTI® HIV self-test is highly accurate, it can have a high invalid rate when persons are given the self-test without additional resources and linkages. (Of note, we were unable to locate any published data on the invalid rate of the INSTI® HIV self-test from other studies in Canada.)

Second, when evaluating effectiveness, one must also consider whether self-testing, as an intervention, yields desired outcomes, which are to identify undiagnosed HIV infections and help people obtain undetectable viral loads. Many assumptions must hold true for self-testing to help achieve the 95-95-95 targets (UNAIDS, 2020). To explain, it is estimated that up to 70% of HIV transmission involves people who are undiagnosed because, once diagnosed, most persons take steps to mitigate transmission (Marks et al., 2005). Starting HIV treatment is one way to reduce onward HIV transmission because an undetectable viral load eliminates the risk of transmission, popularized by the saying *undetectable equals untransmittable* or U = U. The belief is tenuous that self-testing will automatically correspond with linkage to care and U = U (Walensky & Paltiel, 2006). The decreases in transmission seen due to U = U are not merely the result of testing, but of supportive and culturally appropriate and safe care, whereby people want to and can access services, and whereby they can afford, obtain, and take medications (Armoon, et al., 2021). U = U, in other words, materializes when persons living with HIV can and want to access treatment, and are supported as they overcome the stigma associated with their diagnosis. Testing is the entry point to care and should not be the only intervention that receives funding—especially as a one-time investment as Canada has operationalized it currently.

Of further issue regarding effectiveness is that treatment is not the only outcome after HIV testing. Re-testing (whether routinely or outside of window periods) and access to PrEP are also important—and, in fact, likely the most frequently needed interventions. In GetaKit, with (1) 81% of our sample belonging to HIV priority populations and (2) a positivity rate of 0.4%, an average 1 out of every 250 participants had a positive result, while 202 out of every 250 participants had a negative result and had a clinical indication for re-testing and pre-exposure prophylaxis (PrEP) (Tan et al., 2017). Following the principles of status neutral care, in which persons obtain access to appropriate HIV follow-up (treatment or prevention), self-testing should ensure access to efficacious prevention strategies, as it is a key step in achieving the 95-95-95 targets in Canada.

However, despite Canada’s investment in HIV self-tests, only some persons can obtain PrEP, whether due to costs, access to healthcare providers, or abilities to take medication as prescribed. PrEP is expensive and requires prescriptions and clinical monitoring every three months. Considering these barriers, self-testing may inversely exacerbate inequities related to prevention by only providing it to those with a pre-existing awareness of this intervention, or by making it available only to those with sufficient socioeconomic status to purchase the medication. This conflicts with the World Health Organization (2022) declaring that “equalize” is the key word for 2022-2023 for HIV. Perhaps, rather than exclusively focusing on self-testing, Canada should adopt a comprehensive strategy that decreases stigmatization in healthcare and integrates testing with affordable access to treatment and to PrEP for members of HIV priority populations. Instead, Canada opted to “decentraliz[e] testing” to combat “significant barriers to accessing HIV testing, including stigma and discrimination experienced in healthcare settings”. The option Canada selected did not aim to reduce discrimination in healthcare, but rather, required that Canadians change their practices by seeking HIV testing in new ways.

The next two questions from the impact fraction model focus on examining intervention uptake within an appropriate target population. Averaging about 300–350 orders per month in Ontario alone, GetaKit demonstrates a clear demand for self-tests. Similar uptake by another Canadian project (see Rourke, 2021) reinforces that distributing self-tests will likely not be an issue. For targeting, that 81% of GetaKit participants belong to HIV priority populations suggests that the uptake of self-tests is among the correct target populations. However, a key finding in GetaKit is that the US CDC-based (CDC 2021) screening algorithm deems 27% of potential participants ineligible, due, for example, to inadequate risks or too frequent testing, among others. The most recent data available from another HIV self-testing project in Canada, which does not use any form of risk assessment, identified that only 51% of its participants belonged to the groups most affected by HIV (Rourke, 2021). This suggests that access to HIV self-testing without an initial risk assessment may result in upwards of 1 in 2 test kits being used by persons with little to no risk for HIV acquisition—with the consequences of mass screening in low prevalence populations being well established. With a test that is as sensitive and specific as the one used in Canada (estimated sensitivity of 99.8% and specificity of 99.5%) (CATIE, 2020), while there is a 95% positivity predictive value in a population with an estimated HIV prevalence of 11% (as it is among gbMSM in Ottawa) (PHAC, 2011), the positive predictive value drops to 29% in populations with an HIV prevalence of 0.2% (as it is in Canada) (PHAC, 2022b). Increased uptake among low prevalence groups risks wasting resources and subjecting persons to the potential harms of false positive results, including psychological distress and uncertainty as to whether HIV disclosure laws would apply, pending serology to rule out HIV infection. As ASOs are often situated within the communities most affected by HIV, our aim is to use GetaKit’s self-assessment to minimize the risk of false positive results, poor positive predictive values, and the harms of inappropriate HIV self-testing. Ideally, similar outcomes occur for Canada’s program as well. It is also hoped that Canada’s program can provide kits to persons who do not have access to ordering websites (such as via GetaKit.ca), although GetaKit has surmounted this issue by implementing on-site registration (through computer, tablet, or paper) at ASOs in Ontario and via street-outreach.

In closing, while the true effect of mass distribution of HIV self-tests via ASOs in Canada is yet to be seen, our review suggests that this strategy could increase testing, but that it runs the risk of wasting resources without decreasing the proportion of persons with undiagnosed HIV infection. That is, people may test frequently without identifying many new diagnoses. With the screening and eligibility criteria for these tests being unclear, the potential yield of mass self-testing initiatives may be further undermined when low-risk persons use these tests and generate false positive results, including the potential harms of this result. If, however, we do obtain new diagnoses, testing positive is not the end goal. That goal is the elimination of ongoing HIV transmission, which most commonly is achieved by means of established linkage to care. The Government of Canada (PHAC, 2022b) explicitly stated that their rationale for “decentralizing testing [is] to support progress toward ending HIV as a public health concern by 2030”. Canada, instead, appears to have focused on testing and seems to be just hoping that everything else will naturally flow from this. If Canada is serious about achieving the 95-95-95 goals and about actually improving the health of gay, bisexual, trans, and other men who have sex with men; members of Indigenous communities; persons of African, Caribbean, and Black ethnicities; and people who use injection drugs, it might be time for our country to develop and implement a comprehensive plan for HIV, which ensures—as the WHO has called for—equalizing access to treatment and prevention services, both of which continue to remain unattainable for many in Canada, in particular the racialized and sexuality minority groups that continue to be most affected by HIV.

## Data Availability

Data are available upon request.
